# Regulation of NAD^+^ Homeostasis by *Ss*NrtR in *Streptococcus Sobrinus*: A Critical Determinant of Its Cariogenic Potential

**DOI:** 10.3390/pathogens14121213

**Published:** 2025-11-28

**Authors:** Shuojie Lv, Haojie Yu, Dandan Shao, Yuheng Zhao, Jian Chen, Wanying Zheng, Qingjing Wang

**Affiliations:** 1 Key Laboratory of Artificial Organs and Computational Medicine of Zhejiang Province, Key Laboratory of Pollution Exposure and Health Intervention of Zhejiang Province, Shulan International Medical College, Zhejiang Shuren University, Hangzhou 310015, China; 18368021019@163.com (S.L.); cj120325@outlook.com (J.C.); 15757739050@163.com (W.Z.); 2 Stomatology Hospital, School of Stomatology, Zhejiang University School of Medicine, Zhejiang Provincial Clinical Research Center for Oral Diseases, Key Laboratory of Oral Biomedical Research of Zhejiang Province, Cancer Center of Zhejiang University, Hangzhou 310006, China; yhj89@zju.edu.cn; 3 Departments of Clinical Nutrition, The Second Affiliated Hospital of Zhejiang University, Hangzhou 310009, China; ddshao@zju.edu.cn; 4 College of Biology and Environmental Engineering, Zhejiang Shuren University, Hangzhou 310015, China; zyh04122@126.com

**Keywords:** NAD^+^ biosynthesis, *Streptococcus sobrinus*, regulator, ADP-ribose, virulence

## Abstract

Nicotinamide adenine dinucleotide (NAD^+^) serves as a critical cofactor in redox reactions and metabolic transformations catalyzed by NAD-dependent enzymes and is essential for bacterial survival and virulence. The biosynthesis of NAD^+^ in the cariogenic pathogen *Streptococcus Sobrinus* (*S. sobrinus*), a pivotal participant in oral cavities of children and adolescents with a history of caries, has yet to be explored. Bioinformatics, genetics, and biochemical techniques were used to identify NAD^+^ biosynthesis pathways and corresponding regulator in *S. Sobrinus*. *S. sobrinus* lacks de novo NAD^+^ synthesis pathway but comprises NA and Nam salvage pathway I (PncA-PncB-NadD-NadE) and PnuC-NadR salvage pathway III. NiaY and PnuC were involved in the salvage pathways. N-terminal domain of *Ss*NrtR regulator was identified as DNA-binding domain binding to the *pnuC* and *pncB* probe, and addition of ADP-ribose reversed the binding of *Ss*NrtR to the target promoters to regulate NAD^+^ salvage pathways. C-terminal domain of *Ss*NrtR was non-catalytic, consistent with loss of Nudix motif conservation. Furthermore, the abrogation of *nrtR* compromised multiple pathogenic traits, including cellular proliferation, acidogenesis, and the architecture/mechanical integrity of biofilms. Consequently, this mutant exhibited attenuated virulence in a rat caries model. Our findings conclusively demonstrate that *Ss*NrtR-mediated regulation of NAD^+^ homeostasis is a critical determinant of the cariogenic potential of *S. sobrinus*. This study identifies SsNrtR as a previously uncharacterized NAD^+^-responsive regulator that integrates metabolic homeostasis with the control of virulence in Streptococcus sobrinus. These findings elucidate a novel metabolic–virulence regulatory axis in this species and position SsNrtR as a promising target for the development of anti-caries interventions.

## 1. Introduction

Nicotinamide adenine dinucleotide (NAD) engages in a plethora of metabolic and regulatory pathways as a substrate that is consumed during these processes [1]. In bacteria, the primary route for NAD biosynthesis is de novo synthesis from aspartate, which is complemented by various pathways that synthesize NAD from exogenous precursors such as nicotinamide, nicotinic acid, and riboside. The combination of these NAD metabolic pathways varies significantly across different organisms, leading to diverse mechanisms of NAD biosynthesis. Despite the differences in synthetic pathways, the fundamental process remains crucial for NAD production within bacterial systems. In this study, “NAD^+^” specifically refers to the oxidized form of nicotinamide adenine dinucleotide, whereas “NAD” is used as a general term encompassing both oxidized (NAD^+^) and reduced (NADH) forms. NAD^+^ synthesis is controlled by regulatory proteins, such as NadR, NiaR, and NrtR (Nudix-related transcription factors) [2,3]. The nadR gene, identified as the first transcriptional regulator of NAD synthesis in *Salmonella* and *Escherichia coli*, was later found to be a multifunctional protein with two enzymatic activities [4]. The discovery of NadR provides a different example of the formation of a novel type of transcriptional regulator [5]. The second regulator, which is mechanistically different from the first, was discovered and proven in *Bacillus subtilis* [6]. In addition, the third regulator, the NrtR family, designated Nudix-related transcriptional regulators, was identified across various bacterial genomes by analyzing and comparing the locations of sites [7].

*S. sobrinus* is a significant Gram-positive bacterium that is predominantly found colonizing the human tooth surface and is recognized as a major contributor to dental caries in humans. This organism is particularly prevalent in the oral cavities of children and adolescents with a history of caries, where it is present in higher concentrations than in those without caries. Its role in dental health is highly important; however, our understanding of *S. sobrinus* remains limited. The bacterium is known to be associated with the initiation of caries on smooth tooth surfaces and is considered more active in the progression of caries than other species, such as *Streptococcus mutans* [8]. Another species of Streptococcus, *S. mutans*, also shows this pattern, and these two species often appear together clinically. Compared with that of *S. mutans,* the acid production activity of *S. sobrinus* is greater than that of *S. mutans* [9] and in vitro cultivation has also proven that *S. sobrinus* acidifies more than *S. mutans does* [10]. The ability to aggregate into dental-plaque biofilms of *S. sobrinus* via dextran-mediated interactions is also more pronounced.

However, the role and regulation of NAD^+^ in the pathobiology of *S. sobrinus* remain unclear. Accordingly, we investigated the NAD^+^ biosynthesis pathway in *S. sobrinus*, including the nicotinic acid (NA) and nicotinamide (Nam) salvage/recycling pathway (Salvage I) and the PnuC-NadR salvage biosynthesis pathway (Salvage III), which are regulated by the NrtR family regulon, which are SsNrtR regulators. SsNrtR(*S. sobrinus* NrtR) resembles a Nudix-like effector domain, which is inverted, in contrast with the domains of NrtR in *Streptococcus suis* serotype 2 (SS2) reported in our previous study [11]. In a previous study, we demonstrated that the regulatory effect of NrtR influences the NAD^+^ synthesis capability of this bacterium, although it is not the primary regulatory pathway for NAD^+^ synthesis in the zoonotic pathogen *Streptococcus suis* serotype 2 (SS2) [11]. Given the pivotal role of NrtR in maintaining NAD^+^ homeostasis, this prompted us to further investigate its functional characteristics in other bacterial species. This study aimed to test whether *S. sobrinus* NrtR (SsNrtR) functions as a transcriptional repressor that directly regulates NAD^+^ salvage metabolism and thereby contributes to NAD^+^ homeostasis and physiological adaptation in this cariogenic species. We applied a functional analysis of the NrtR protein from the cariogenic bacterium *S. sobrinus*, which is anticipated to modulate the salvage and recycling of NA/Nam and NRam by binding to the promoter region of *pncB* and the *pnuC-nadR* operon.

## 2. Materials and Methods

### 2.1. Bacterial Strains and Cultivation Conditions

In this research, the bacterial strains utilized were *Escherichia coli* and *Streptococcus sobrinus* ATCC 33478 [12,13]. The *Escherichia coli* cultures were cultivated in Luria–Bertani (LB) broth, which is composed of 10 g/L sodium chloride, 10 g/L tryptone, and 5 g/L yeast extract in distilled water; solid medium supplemented with 2 g agar per 100 mL at 37 °C with agitation, unless otherwise stated. In contrast, for *Streptococcus sobrinus* ATCC 33478, sterile CDM medium containing 1% glucose was used for cultivation under anaerobic conditions at 37 °C. For solid cultivation, THY medium with 1.5% agar was used, antibiotics were selected with 400 μg/mL spectinomycin, and the mixture was incubated under anaerobic conditions at 37 °C for 24 h [14]. Bacterial growth was assessed through OD600 measurements throughout the cultivation process.

To obtain protein in large quantities, monoclonal techniques, which include polymerase chain reaction (PCR), purification, enzyme digestion, ligation, and transformation, have been used. This technology first uses PCR technology through continuous amplification to quickly and specifically obtain trace samples, and then, through enzyme digestion and ligation, the target gene fragment is connected to the plasmid to construct a plasmid containing the target gene fragment *SsnrtR*. This ligation product was then transferred into DH5a to amplify the *Escherichia coli* containing the plasmid of the target fragment. After plasmid extraction, the gene was transferred into BL21 for protein amplification. This protein can meet the purpose of cloning, and through protein cloning and purification, many proteins (enzymes) can be obtained.

### 2.2. Generation of Gene Knockout Mutants and Functional Complementation

To elucidate the function of SsNrtR in *Streptococcus sobrinus*, an SsNrtR knockout mutant was generated using homologous recombination. Two flanking regions of the target gene-approximately 1 kb each-were amplified from logarithmic-phase *S. sobrinus* genomic DNA using specific primers. Additionally, a spectinomycin resistance cassette of roughly 1 kb was amplified from plasmid pDL278. All three fragments were digested with restriction enzymes, purified by gel electrophoresis, and assembled via overlap extension PCR with primers annealing to the extremities of the upstream and downstream regions. This produced a recombinant fragment approximately 3 kb in length. *S. sobrinus* was grown overnight, diluted 1:100 in THY medium supplemented with 5% horse serum, and incubated anaerobically at 37 °C for 2 h. Synthetic competence-stimulating peptide (XIP 0.8 µg/mL) was then added, followed by another 30 min of incubation. The recombinant fragment was introduced, and transformation proceeded for 3 h under the same conditions. Transformants were selected on THY agar plates containing spectinomycin, ensuring the incorporation of the resistance cassette and deletion of SsNrtR. Chromosomal mutagenesis was performed as previously established. For functional complementation, the nrtR coding sequence along with its native promoter was amplified with primers CnrtR-F and CnrtR-R and cloned into the E. coli–S. sobrinus shuttle vector pVA838, yielding plasmid pVA838::nrtR. This construct was used for subsequent complementation assays.

### 2.3. Protein Expression, Purification, and Characterization

Recombinant E. coli BL21(DE3) cells were cultured to mid-log phase and expression was triggered by adding IPTG at the optimal concentration. After harvesting, the cell pellet was resuspended in 20 mM imidazole buffer and lysed by 80 min sonication on ice. The lysate was clarified by two successive centrifugation steps (18,000 rpm, 1 h, 4 °C), retaining the supernatant each time. The cleared extract was loaded onto a Ni^2+^-NTA column connected to an ÄKTA chromatography system (General Electric Company, Boston, MA). Bound protein was eluted with a stepwise imidazole gradient (20–500 mM). Fractions containing the desired protein were pooled and assessed for purity by Coomassie-stained 12% SDS-PAGE.

### 2.4. Electrophoretic Mobility Shift Assay

To elucidate the molecular recognition between the target protein and its corresponding DNA sequence, an electrophoretic mobility shift assay (EMSA) was employed. This assay hinges on the annealing of two complementary oligonucleotides in a TEN buffer solution comprising 10 mM Tris-HCl, 1 mM EDTA, and 100 mM NaCl at pH 8.0 to synthesize DNA probes that are specifically recognized by the target protein. These probes were subsequently mixed with the target protein that had been subjected to purification in EMSA buffer, and the mixture was equilibrated at room temperature for 150 min. The resulting protein—DNA complexes were then resolved through polyacrylamide gel electrophoresis at an 8% concentration. The DNA bands, which exhibited a migration shift as a consequence of protein interaction, were subsequently revealed by staining with DNA loading buffer, thus facilitating the visualization of the shifted bands.

### 2.5. Assays of Enzymatic Activity

An HPLC-based assay, as detailed in reference [15], was employed to quantify the Nudix hydrolase activity of NrtR proteins. The assay mixture consisted of 50 mM HEPES buffer at pH 8.0 supplemented with either 5 mM MgCl_2_, 1 mM DTT, and 1 mM nucleoside diphosphate derivatives as substrates, along with varying concentrations of purified recombinant proteins. Following a 30-min incubation at 37 °C, reactions were terminated with HClO_4_.

### 2.6. β-Galactosidase Activity Assays

ONPG was dissolved in peptone water-supplemented buffer and sterilized. The solution was then aliquoted into 0.5 mL tubes and sealed. A loopful of a log-phase bacterial mixture was inoculated into the aliquots, followed by incubation at 37 °C. The development of a yellow color within 1–3 h indicated β-galactosidase (β-gal) production, while the absence of color change after 24 h was interpreted as a negative result.

### 2.7. Quantification of Intracellular NAD^+^ and NADH Levels

Cells in the logarithmic growth phase were centrifuged at 10,000× *g* and 4 °C for 10 min. The resulting pellet was resuspended in lysis buffer and incubated at room temperature for 15 min to promote lysis. The suspension was then sonicated for one minute to ensure complete disruption. After a second centrifugation step under the same conditions (10,000× *g*, 4 °C, 10 min), the supernatant was collected for quantification of NAD^+^ and NADH using the Amplite Colorimetric Assay Kit, in strict accordance with the manufacturer’s protocol. All steps were performed in triplicate in independent experiments.

### 2.8. Measurement of Biofilm Biomass

Cultures of WT and mutant strains were grown to mid-exponential phase (OD_600_ = 0.5), diluted 1:100, and aliquoted into 96-well plates. After incubation at 37 °C for 12 and 24 h, non-adherent cells were removed by gently washing the biofilms with sterile distilled water. The adherent biofilms were air-dried briefly, stained with 200 µL of 1% aqueous crystal violet for 30 min at room temperature, and then rinsed twice with water. Following drying, the bound dye was solubilized using 200 µL of ethanol per well over 30 min. A 100 µL aliquot of the solubilized solution was transferred to a new microplate, and the absorbance was measured at 570 nm using a spectrophotometer (Eon Microplate, BioTek Instruments, Winooski, VT, USA).

### 2.9. Assessment of Acid Production in S. sobrinus

To evaluate the acid production capacity of the wild-type (WT), mutant Δ*nrtR* (*nrtR* gene deletion mutant of *Streptococcus sobrinus*), and complementary strain CΔ*nrtR*(the Δ*nrtR* mutant complemented with a functional *nrtR* gene), brain heart infusion (BHI) broth supplemented with 5% sucrose was prepared at pH values ranging from 4.0 to 7.0 in 0.5 increments. Bacterial suspensions of each strain were inoculated at a 1:10 (*v*/*v*) ratio into the medium and incubated anaerobically at 37 °C under a gas mixture of 80% N_2_, 10% H_2_, and 10% CO_2_ for 24 h. The pH was monitored and recorded at regular intervals throughout the incubation period. All experiments were performed in triplicate, and mean values were calculated.

### 2.10. In Vivo Caries Model in Rats: Induction by Cariogenic Challenge

For the animal model component of this study, a cohort of 23-week-old female Specific Pathogen Free (SPF) Wistar rats was ethically sourced from Hangzhou Hengshi Biology (Hangzhou, China), with the breeding and all experimental procedures involving these animals conducted under the explicit approval of the Animal Ethics Committee at Zhejiang Shuren University. The study’s adherence to the European Community guidelines for the care and use of laboratory animals (Directive 2010/63/EU) ensured the welfare and ethical treatment of the animals throughout the experimental process. Within the context of establishing a dental caries model, the rats were initially allowed a three-day acclimatization period to adjust to their new dietary and environmental conditions, a step crucial for their well-being and for minimizing stress prior to the commencement of the experimental procedures. They were then randomly divided into 4 groups (n = 5/group): the normal control group (N), WT group, ΔnrtR group, and CΔnrtR group. The rats were fed antibiotics (ampicillin, 1.0 g/kg diet) for 5 consecutive days and then challenged with 2 × 10^9^ colony-forming units (CFUs) of S. sobrinus 3 times (once a day).

In the current investigation S. sobrinus, which is known to play a pivotal role in dental caries, was initially inoculated in Brain Heart Infusion (BHI) broth at a dilution ratio of 1:1000. This inoculation was followed by an incubation period at 37 °C, designed to promote the bacteria’s growth to the logarithmic phase. Subsequently, under anaerobic conditions that are conducive to S. sobrinus’s growth, the culture was transferred to a 13 mL shake tube filled with BHI broth, and the tube was securely sealed to maintain the required environment. After the incubation period, the bacterial cells were harvested, and a homogeneous suspension was prepared by complete resuspension in 200 μL of PBS, ensuring a uniform distribution of the bacteria for further analysis. The optical density (OD600) at 600 nm was meticulously recorded, serving as a quantitative measure of the bacterial concentration. A bacterial suspension with an OD600 of 1, which indicates a specific bacterial density, was selected for inoculation onto solid Mueller-Hinton blood (MSB) agar. The plates were then incubated under anaerobic conditions at 37 °C for 48 h, a duration sufficient to allow for the growth and subsequent enumeration of colony-forming units (CFUs).

All animal experiments were conducted in the laboratory at Zhejiang Shuren University. All procedures were conducted in accordance with the Guide for the Care and Use of Laboratory Animals by the National Research Council of the United States. The details of animal housing, bacterial colonization, and euthanasia procedures are provided in Supplementary Materials S2. Cervical dislocation surgery was performed, followed by excision of the mandible and removal of excess tissue to assess the bacterial levels present and the extent of caries formation. All mandibles were fixed in 4% paraformaldehyde solution and stained with 0.4% purple urea ammonium for 12 h. After evaluating smooth surface caries, the main grooves of each tooth were exposed through mesiodistal sagittal sectioning. Imaging of the grooves and smooth surfaces of all mandibles was performed using a stereo microscope (EZ4W, Leica, Wetzlar, Germany). The enamel caries (E), superficial dentin caries (Ds), and medium dentin caries (Dm) of each rat were scored and evaluated under a microscope according to the Keyes scoring criteria [16]. Detailed descriptions of the Keyes caries scoring system, together with procedures for ethical animal maintenance and health surveillance, are included in Supplementary Material S2.

### 2.11. Bioinformatic Analysis

NrtR homologs from different species were identified via NCBI protein BLAST (BLASTP; https://blast.ncbi.nlm.nih.gov/Blast.cgi?PAGE=Proteins/, accessed on 30 October 2025). Multiple alignments of either NrtR proteins or NrtR-binding sites were carried out via the ClustalW2 program (http://www.ebi.ac.uk/Tools/clustalw2/index.html, accessed on 30 October 2025), and the final output file was processed via the ESPript 2.2 server https://espript.ibcp.fr/ESPript/ESPript/index.php, accessed on 30 October 2025) [11]. Structural modeling of *Ss*NrtR was performed with SWISS-MODEL (https://www.swissmodel.expasy.org/, accessed on 30 October 2025). SPSS Statistics 22.0 (SPSS, Inc., Chicago, IL, USA) was used for data analysis. All the data are presented as the means ± SDs. When applicable, ANOVA/2-tailed Student’s *t* test was applied to analyze the differences between groups. A *p* value < 0.05 was defined statistically significant, and a *p* value < 0.01 was indicate a high level of statistical significance.

## 3. Results

### 3.1. Verification of the NAD^+^ Biosynthesis Pathway in S. sobrinus

**Figure 1:**
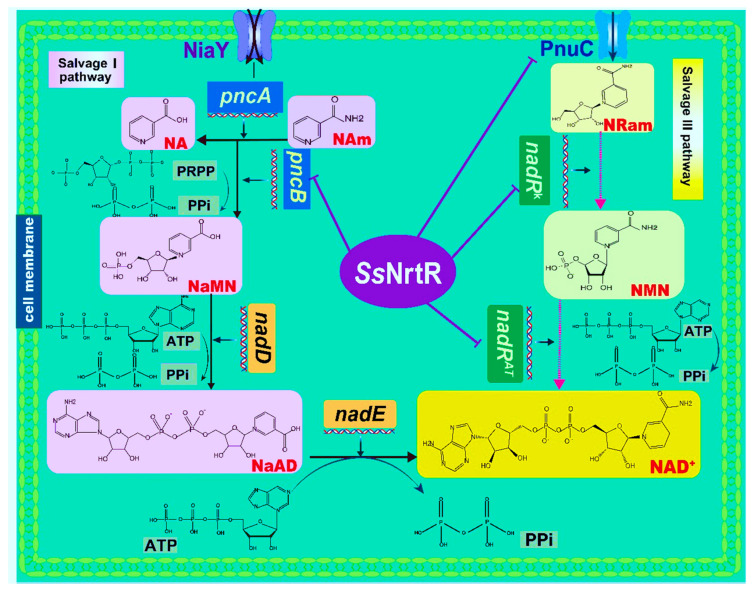
NAD^+^ biogenesis and its transcriptional regulation in *S. sobrinus.* Pathway diagram showing biochemical transformations (arrows) of precursors and intermediates (shown via the same abbreviations as in the text). In the Nam salvage pathway (salvage I pathway), NaMN is synthesized from NA and Nam precursors that are taken up by the niacin transporter (NiaY). The Universal NaMN-to-NAD pathway utilizes nicotinate mononucleotide adenylyltransferase (*nadD*) and NAD synthetase (*nadE*). In the salvage III pathway, NAD^+^ is synthesized from the exogenous RNam precursor delivered by the RNam transporter (*pnuC*) via consecutive reactions catalyzed by two separate domains of NadR, nicotinamide mononucleotide adenylyltransferase (*nadR^AT^*), and nicotinamide riboside kinase (*nadR^K^*). The purple lines point to regulated genes (NrtR regulon members).

Comparative genomic analysis revealed a lack of de novo NAD^+^ biosynthesis genes in *S. sobrinus* while the salvage I pathway and salvage III pathway were present (Figure 1). The Salvage I pathway includes the NiaY transporter, which facilitates the cellular uptake of NA and Nam, which are converted to NaMN by PncA and PncB, and nicotinamide deamination and nicotinate phosphoribosyltransferase activities, respectively. Subsequently, NAD^+^ is generated via nicotinate mononucleotide adenylyltransferase (*nadD*) and NAD synthetase (*nadE*), which are encoded by the *nadDE* operon (Figure 1). The Salvage II pathway, which is pivotal for NAD biosynthesis, employs the enzymes nicotinamide phosphoribosyltransferase (*nadV*) and nicotinamide mononucleotide adenylyltransferase (*nadM^AT^)*. *nadV* initiates this process by converting nicotinamide into nicotinamide mononucleotide (NMN), which is then further transformed into NAD^+^ by *nadM^AT^*, thereby contributing to the cellular NAD pool [17]. In the third salvage pathway, NAD^+^ synthesis is facilitated by the exogenous nicotinamide riboside (NR) precursor, which is transported into the cell by the NR transporter PnuC. This process involves a series of reactions catalyzed by distinct domains of the NadR enzyme. Initially, the nicotinamide riboside kinase (*nadR^K^*) domain phosphorylates NR to form NMN, and then the nicotinamide mononucleotide adenylyltransferase (*nadR^AT^*) domain catalyzes the conversion of NMN into NAD (Figure 1) [18].

### 3.2. Detection of NrtR Regulator in S. sobrinus

In *S. sobrinus*, the NAD^+^ salvage III pathway, including an operon that encodes putative orthologs of *pnuC-nadR* and an NrtR regulator (*Ss*NrtR), was identified. NrtR-binding sites are located in the *pnuC-nadR* operon and *pncB* gene promoter region (Figure 1), indicating that NAD^+^ salvage pathways in *S. sobrinus* are regulated by the NrtR homolog *Ss*NrtR protein. To determine the role of *Ss*NrtR, the recombinant *Ss*NrtR protein, which was fused with a hexa-histidine tag, was extracted from *E. coli* BL21 (DE3) strains containing the pET28b-*nrtR* plasmid. The purification involved nickel NTA agarose affinity chromatography and subsequent gel filtration using a Superdex 200 column.

**Figure 2:**
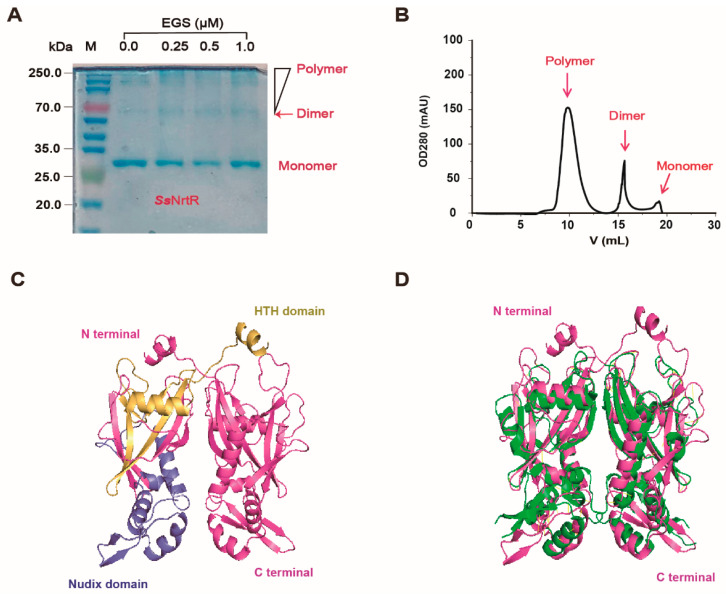
Biochemical characteristics of the SsNrtR protein. (**A**) The molecular weight and purity of the purified *Ss*NrtR protein in the 10% SDS-PAGE mixture. EGS-based chemical cross-linking assays suggested that *Ss*NrtR forms a dimer. The protein samples were separated via gel filtration with Superdex 200 (GE Healthcare). (**B**) FPLC profile of the purified protein *Ss*NrtR, indicating its homogeneity and the presence of the monomer, dimer and polymer forms. (**C**) Ribbon illustration of the modeled structure of the *Ss*NrtR protein. (**D**) Structural alignment of NrtR proteins in *S. sobrinus* (*Ss*NrtR, in magenta) and *Shewanella oneidensis* (*SoNrtR*, in green). The *Ss*NrtR protein contains two domains: the N-terminal wHTH DNA-binding domain (in yellow) and the C-terminal Nudix domain (in slate).

**Figure 3:**
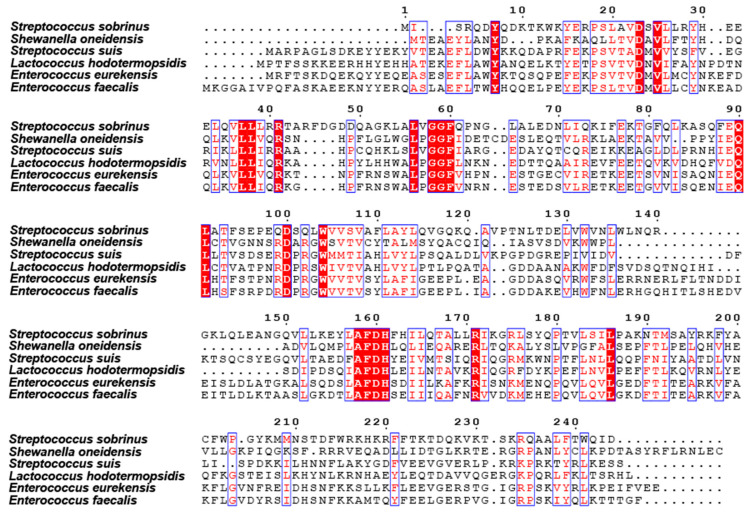
Multiple sequence alignment of NrtR with homologs from other species. The protein sequences of the NrtR homologs were aligned via ClustalW2. The resulting photographs were generated via the ESPript 2.2 program. Identical residues are indicated with white letters on a red background. Similar residues are illustrated in black letters with a yellow background. Varied residues are in black letters, and dots represent gaps.

The purified protein was then analyzed for homogeneity via 10% SDS-PAGE (Figure 2A). The recombinant protein sample was highly pure, with an intense band at approximately 28 kDa, which was consistent with the expected molecular weight (Figure 2A). Here, we observed that the protein tended to form dimers. We confirmed this by EGS cross-linking, which revealed that *Ss*NrtR forms dimers, oligomers, and multimers, with monomer levels decreasing as the EGS concentration increased (Figure 2A). The UV absorbance at OD280 during gel filtration further corroborated these findings (Figure 2B). Sequence analysis indicated that *Ss*NrtR comprises an N-terminal winged helix-turn-helix (wHTH) domain for DNA binding and a C-terminal domain that is characterized by hydrolytic enzyme activity and is homologous to ADP-ribose (ADPR) pyrophosphatases of the Nudix family (Figure 2C). Structural alignment of NrtR proteins in *S. sobrinus* (*Ss*NrtR, in magenta) and in *Shewanella oneidensis* (*So*NrtR, in green) highlights the similarity in protein spatial structure and functional domains (Figure 2D). The *Ss*NrtR protein has significant sequence conservation with its counterparts in various streptococcal species [11] and other distantly related organisms (Figure 3).

### 3.3. Identification of the Function of the N-Terminal Domain in the SsNrtR Regulon

**Figure 4:**
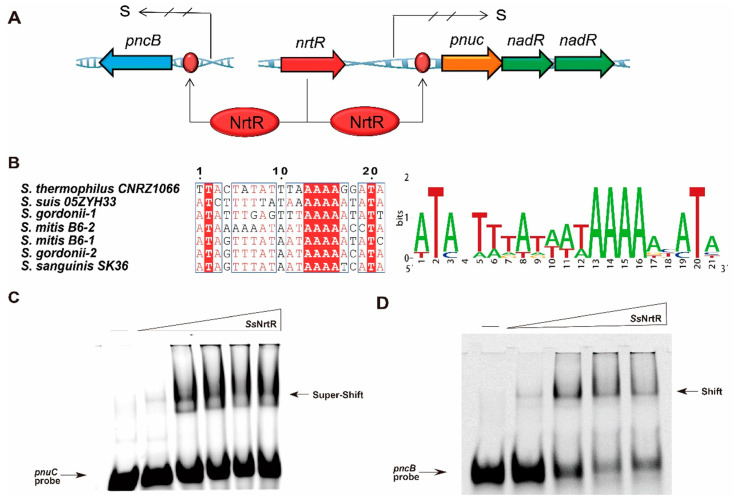
Identification of the N-terminal wHTH DNA-binding domain in the *Ss*NrtR protein. (**A**) Genetic context of *nrtR* and its regulatory target genes (*pnuC*, *nadR*, *pncB*) in *S. sobrinus*. (**B**) The sequence of the conserved NrtR-recognizable motif in Streptococcaceae. (**C**) *Ss*NrtR binds to the *pnuC* probe in a dose-dependent manner. (**D**) *Ss*NrtR bound to the *pncB* probe in a dose-dependent manner. A 200 bp DNA probe encompassing the *pnuC*/*pncB* gene promoter region was obtained by annealing the primers *pnuC*-F/R and *pncB*-F/R (Supplemental Table S3). The arrow indicates the shifted/supershifted DNA-protein complex.

The NrtR protein from *S. sobrinus* was incubated with 200 bp DNA fragments that encompass the predicted binding sites within the *pncB* and *pnuC* promoter regions (Figure 4A), and it was observed that Streptococcus species, including *S. sobrinus*, possess 1 to 2 NrtR-binding sites within their genomes (Figure 4B). An electrophoretic mobility shift assay revealed a dose-dependent shift, even a supershift, confirming that *Ss*NrtR bound to the specific *pnuC* probe (Figure 4C) to also regulate the *nadR* gene since the two genes are organized into an operon (*pnuC-nadR* operon). An analogous shift was observed in the *pncB* probe (Figure 4D), suggesting that the *Ss*NrtR regulon can regulate the NAD^+^ salvage I pathway (NA salvage pathway) and salvage III biosynthesis pathway (Pnuc-NadR pathway) via its N-terminal DNA-binding domain.

### 3.4. Binding of SsNrtR to Target Genes Can Be Impaired by ADP-Ribose

**Figure 5:**
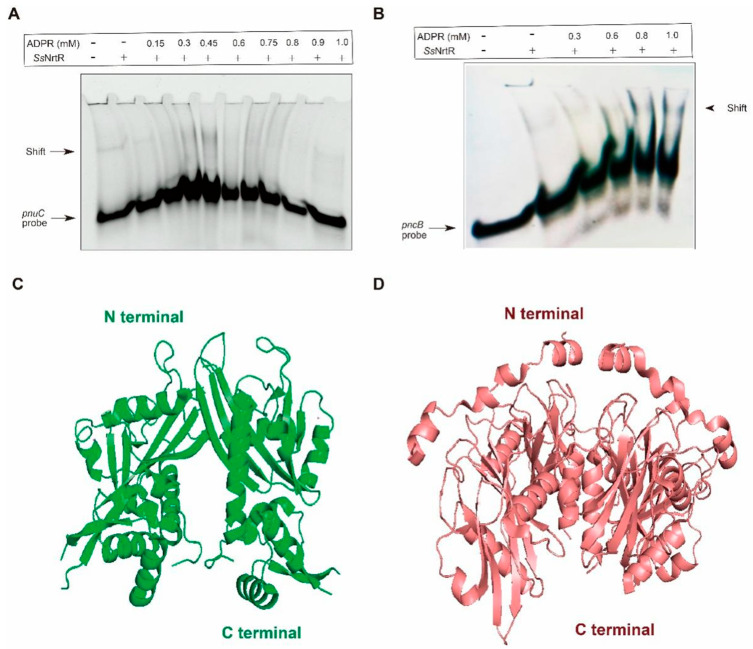
The binding of *Ss*NrtR to target genes can be reversed by ADPR. (**A**) Addition of ADPR reversed the binding of *Ss*NrtR to the *nrtR* promoter. (**B**) Addition of ADPR reversed the binding of *Ss*NrtR to the *pncB* promoter. (**C**) Crystal structure of the *So*NrtR protein without ADPR. (**D**) Crystal structure of the NUDIX domain of *So*NrtR complexed with ADP ribose. ADPR was added at different concentrations denoted by rectangles (0.0, 0.15, 0.3, 0.45, 0.6, 0.9 and 1.0 mM). Proteins were added at different concentrations denoted by rectangles (0.0, 0.2, 0.6, 1.0, and 2.0 mM). A 200 bp DNA probe encompassing the *pnuC*/*pncB* gene promoter region was obtained by annealing the primers *pnuC*-F/R and *pncB*-F/R (Supplemental Table S3).

ADPR, a key intermediate in NAD^+^ catabolism, has been identified as an essential regulatory mechanism in *Streptomyces species* and plays a critical role in their cellular metabolism and signaling pathways [19]. To explore the role of ADPR in *Ss*NrtR regulation, ADPR was introduced at various concentrations. The protein concentrations were also varied. A 200 bp DNA fragment containing the *pnuC/pncB* gene promoter was synthesized by combining *pnuC*-F/R and *pncB*-F/R. The EMSA data revealed that the interaction between *Ss*NrtR and the *pnuC/pncB* DNA probe was disrupted by ADPR in a concentration-dependent manner (Figure 5A,B), in accordance with the phenomena observed in *S. suis* [11].

Upon examination of the crystal structures of *Shewanella oneidensis* NrtR (*So*NrtR) in the absence (Figure 5C) and presence of ADP-ribose (Figure 5D), the binding of ADPR induces significant conformational alterations in multiple regions of *So*NrtR(Figure 5D), which is termed a “Nudix switch” motif that is likely crucial for the allosteric modulation of DNA binding affinity and the repressor activity of NrtR [20]. Significant conformational shifts are typically observed in this domain following ligand binding, facilitating the approximation of aspartate residues to the substrate. In the presence of ADPR, the wHTH domain may need to assume a subtly distinct conformation, potentially impairing its DNA binding capacity, given that the precise alignment in *Ss*NrtR is essential for establishing a continuous interface that complements the DNA double helix.

### 3.5. Investigation of the C-Terminal Nudix Domain of the SsNrtR Regulon

**Figure 6:**
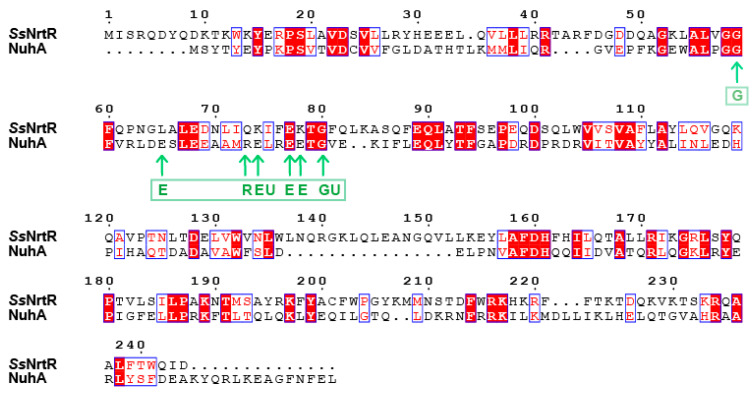
Sequence alignment of the *Ss*NrtR and NuhA proteins. The protein sequences of the NrtR homologs were aligned via ClustalW2. The resulting photographs were generated via the ESPript 2.2 program. Identical residues are indicated with white letters on a red background. Similar residues are illustrated in black letters with a yellow background. Varied residues are in black letters, and dots represent gaps. The amino acids indicated by the arrows are the Nudix motif GX5EX7REUXEEXGU, where U is a hydrophobic amino acid, usually Ile, Leu, or Val.

**Figure 7:**
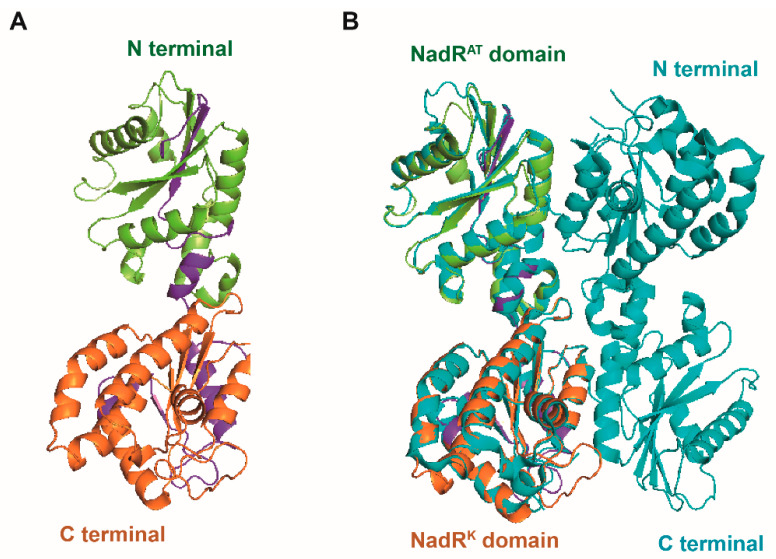
Structural characteristics of the SsNadR protein. (**A**) Ribbon illustration of the modeled structure of the *Ss*NadR protein. (**B**) Structural alignment of NadR proteins in *S. sobrinus* (*Ss*NadR, in limon and orange) and *Lactococcus lactis* (*LlNrtR*, in navy blue). The *Ss*NadR protein contains two domains: the N-terminal nicotinamide mononucleotide adenylyltransferase domain (*nadR^AT^*, in limon) and the C-terminal icotinamide riboside kinase domain (*nadR^K^*, in orange).

The C-terminal domain of *Ss*NrtR is homologous to the ADPR pyrophosphatase from the Nudix hydrolase family. To explore the hydrolase activity of *Ss*NrtR, an HPLC-based assay was performed, and no enzymatic activity was detected under the tested conditions, which is consistent with the report by Rodionov et al. [20]. In contrast to catalytically active Nudix hydrolases, a significant number of NrtR family members may have preserved their enzymatic function. This hypothesis is grounded in the observed absence of conservation within the signature motif GX5EX7REUXEEXGU (Figure 6), a sequence that is invariantly preserved in all known active Nudix hydrolases [21]. However, the NuhA protein, which possesses an intact Nudix motif (Figure 6), exhibited robust ADPR pyrophosphatase activity [22]. Within the majority of NrtR family members, including NrtR in *S. sobrinus*, some conserved signature residues are substituted in a somewhat indiscriminate manner (Figure 6 and Figure 7). According to our previous findings, through targeted mutagenesis of the SSU05_1971 gene, the restoration of the canonical Nudix signature motif contributed to the recovery of the catalytic activity of NrtR in *Streptococcus suis* [11]. These observations imply that the integrity of the Nudix signature motif in NrtR proteins is associated with their enzymatic function.

### 3.6. Regulatory Role of SsNrtR in Bacterial Metabolism

**Figure 8:**
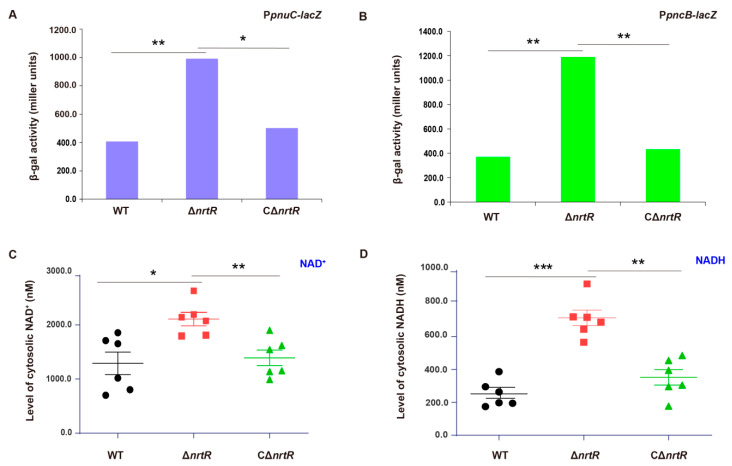
In Vivo validation of NrtR-mediated repression of the NAD^+^ salvage pathways. (**A**) NrtR represses transcription of the *pnuC* operon during mid-logarithmic phase. (**B**) Transcriptional repression is abolished in the Δ*nrtR* mutant, resulting in upregulation of the *pncB* in stationary phase. (**C**) Intracellular NAD^+^ is elevated in the Δ*nrtR* mutant. (**D**) SsNrtR negatively regulates NADH accumulation. Data are presented as mean ± SD from three independent experiments. β-Galactosidase activity and NAD^+^ levels were analyzed by one-way ANOVA followed by Tukey’s test. * *p* < 0.05, ** *p* < 0.01, *** *p* < 0.001.

To determine whether NrtR transcriptionally regulates NAD^+^ salvage pathways in *S. sobrinus*, we constructed the Δ*nrtR* mutant via allelic exchange and a corresponding complemented strain, CΔ*nrtR*. To assess the transcriptional regulation of the *pnuC* and *pncB* promoters by *Ss*NrtR, the *lacZYA* reporter fusion plasmid, pVA838-P*pnuC/*pVA838-P*pncB*, were constructed (Supplemental Table S2). The plasmids were transformed into the wild-type (WT), Δ*nrtR* mutant, and complemented CΔ*nrtR* strains. β-Galactosidase activity assays revealed that *pnuC* and *pncB* expression were significantly elevated in the Δ*nrtR* mutant compared to the WT during both mid-logarithmic and stationary growth phases and the difference was statistically significant (*p* < 0.01), while expression in the complemented strain were restored to WT levels(Figure 8A,B). Consistent with this deregulation, direct quantification showed a 2–3-fold increase in cytosolic NAD^+^ and NADH concentrations in the Δ*nrtR* mutant relative to the parent strain (Figure 8C,D). Intracellular NAD^+^ levels were significantly elevated in the ΔnrtR mutant compared to the wild-type (*p* < 0.05), whereas NADH concentrations in the ΔnrtR mutant showed a marked increase relative to the wild-type (*p* < 0.001). These in vivo findings corroborate the EMSA results (Figure 4C,D), demonstrating that *Ss*NrtR acts as a transcriptional repressor of the NAD^+^ salvage pathways, PncA-PncB-NadD-NadE salvage I and PnuC-NadR salvage III pathways and plays a critical role in maintaining NAD^+^/NADH homeostasis in *S. sobrinus*.

### 3.7. SsNrtR Modulates the Pathogenic Potential of S. sobrinus

**Figure 9:**
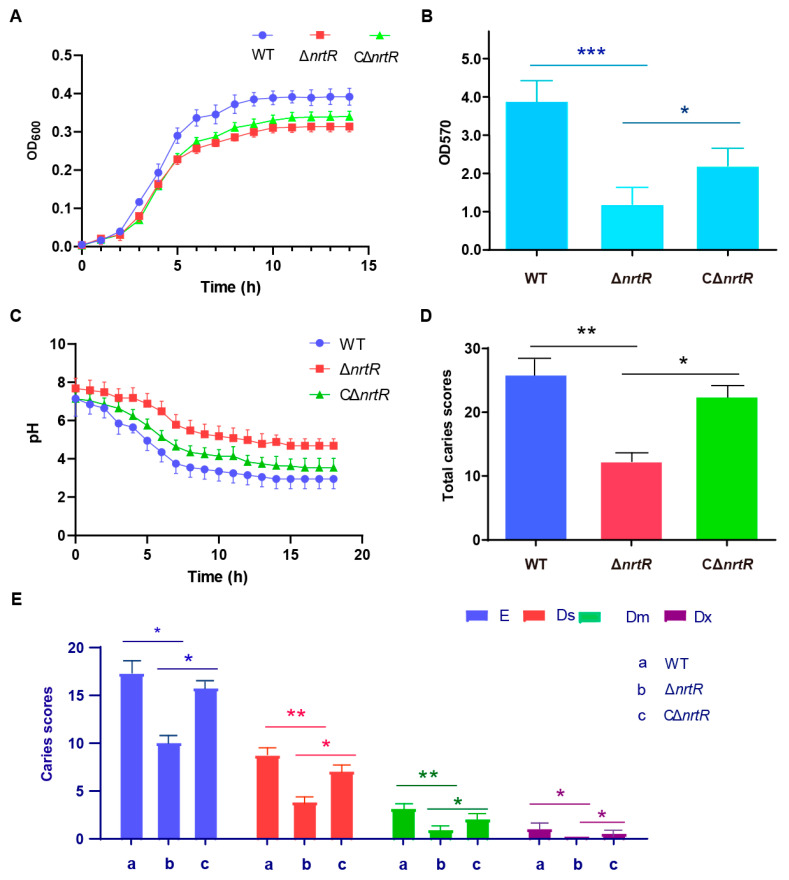
Function of NrtR in the Pathogenicity of *S. sobrinus.* (**A**) Growth curve measurement of WT, Δ*nrtR*, and CΔ*nrtR* strains. (**B**) Biofilm biomass of the WT, Δ*nrtR*, and CΔ*nrtR* strains. (**C**) Acid production by the WT, Δ*nrtR*, and CΔ*nrtR* strains. (**D**) Caries severity in rats infected with the WT, Δ*nrtR*, and CΔ*nrtR* strains. (**E**) Keyes caries scores for different treatment groups. Data are presented as mean ± SD from three independent experiments. Statistical significance was determined by one-way ANOVA with Tukey’s post hoc test. * *p* < 0.05, ** *p* < 0.01, *** *p* < 0.001.

In light of the multifaceted roles of NrtR in bacterial physiology, we assessed a range of potential phenotypes associated with the Δ*nrtR* mutation. A significant reduction in the growth rate of the Δ*nrtR* strain was observed in *S. sobrinus* (Figure 9A). Considering the critical role of dental plaque biofilm in the cariogenicity of *S. sobrinus*. We next assessed biofilm formation in the WT, Δ*nrtR* mutant, and complemented (CΔ*nrtR*) strains. Biofilm formation was significantly reduced in the Δ*nrtR* mutant compared to both the WT and complemented strains (Figure 9B). Biofilm biomass was significantly increased in the ΔnrtR mutant compared to the wild-type strain (*p* < 0.001). As acid production and acid tolerance are essential for the cariogenicity of bacteria, we characterized the relevant properties of the bacteria. The results indicated that The Δ*nrtR* mutant exhibited impaired acid production compared to the WT and complemented strain (CΔ*nrtR*) (Figure 9C).

Additionally, a rat caries model was employed to assess the influence of the *nrtR* gene on bacterial pathogenicity. Throughout the experimental period, all animals exhibited normal behavior, maintained healthy coat conditions, and produced well-formed stools without signs of diarrhea. Body weights were monitored regularly, and no mortality occurred in any group. Furthermore, no statistically significant differences in weight gain were observed between the Δ*nrtR* group and the other groups, suggesting that the genetic alteration did not compromise the overall health of the rats (*p* > 0.05).

To quantify caries development, the molar surfaces—including buccal, lingual, proximal, and occlusal aspects—were divided into discrete carious units according to the criteria detailed in Table 1. Caries lesions were identified and scored as described in the same table, with results provided in Table 2. Using the Keyes scoring system, all 12 molars (both maxillary and mandibular) from each group were evaluated across four lesion types: enamel (E), superficial dentin (Ds), moderate dentin (Dm), and deep dentin (Dx) caries. The Δ*nrtR* mutant group displayed significantly lower caries scores across all four categories compared to the wild-type (WT) group, indicating a pronounced reduction in caries severity (Table 2; Figure 9D,E). Compared with the WT group, the E-type and Dx-type caries scores in the ΔnrtR group were significantly reduced (*p* < 0.05), while the Ds- and Dm-type scores showed highly significant reductions (*p* < 0.01). The complemented strain (CΔ*nrtR*) showed intermediate levels of caries, with scores falling between those of the mutant and WT groups (Table 2; Figure 9D,E). Taken together, these results imply that *Ss*NrtR contributes to the pathogenic potential of *S. sobrinus* during infection.

## 4. Discussion

The enzymes involved in the final steps of NAD biosynthesis, NadD and NadE, are conserved and essential in a wide range of bacterial species and have been identified as promising prognostic markers and potential targets to increase therapy efficacy [23]. NrtRs have been identified in a broad spectrum of bacteria that lack NadR and NiaR [3]. Through comparative genomics, the DNA sequences to which NrtR binds, referred to as NrtR boxes, have been discovered, which aids in the computational modeling of NrtR regulons. These regulons mainly encompass genes related to diverse facets of NAD^+^ metabolism. The N-terminal wHTH domain of *Ss*NrtR has been identified as the DNA-binding domain. The C-terminal Nudix hydrolase domain, however, has lost its catalytic activity, as several residues within the active site of the Nudix motif and L9 loop regions have lost their conservation [3], whereas the domains of NrtR in SS2 have been inverted [11]. This has permitted a plausible evolutionary scenario for the NrtR family. This hypothesis involves the integration of an ADPR--specific Nudix hydrolase with a DNA-binding domain, resulting in the emergence of a transcriptional regulator [3]. It is conceivable that NrtR proteins may have subsequently lost hydrolase activity, which became dispensable due to the presence of other operational Nudix hydrolases. This inference is supported by the substantial and expanding catalog of transcription factors that have been evolutionarily ‘engineered’ to adhere to this paradigm [5,18,24,25].

NrtR functions as a transcriptional repressor that is regulated by ADP-ribose. The hypothesis that ADPR functions as an antirepressor in the regulation of NAD^+^ biosynthesis is grounded in the premise that the cellular accumulation of ADPR could be perceived as an indicator to restore NAD^+^ levels. This rationale is well known, considering that the sole intracellular source of ADPR is the enzymatic hydrolysis of NAD^+^. The interaction of ADPR with the Nudix domain of NrtR facilitates the release of NrtR from its DNA-binding sites, thereby alleviating the repression of NAD^+^ biosynthetic genes. Moreover, ADPR may increase NAD biosynthesis by acting as a precursor for the generation of Rib-P, a necessary component for PRPP, through its hydrolytic cleavage by Nudix enzymes. It seems that the ancestral Nudix hydrolase domain was coopted by NrtR to serve as a signaling module. During this evolutionary adaptation, the domain may have relinquished its catalytic function while retaining the ability to bind ADPR, a product of NAD glycohydrolysis. This binding allows regulation by ADPR through the “Nudix switch,” which transmits the ADPR-binding signal to the DNA-binding domain.

Metabolic processes involving NAD^+^ are associated with the pathogenic potential of certain bacterial species, including Pseudomonas and Actinomyces [26,27,28]. The NrtR mutant strain disrupted the virulence of *Pseudomonas aeruginosa* TBCF10839 in a murine acute airway infection model and limited its metabolite harmony. When cocultured with other isogenic mutant strains, the *nrtR* knockout strain was the most compromised in terms of competitive fitness in vitro in rich media or in vivo within the murine airways l [15,29,30]. This example illustrates how metabolism and virulence are intricately intertwined through key elements of metabolic context.

Research shows When both *S. sobrinus* and *S. mutans* are present simultaneously, extensive carious lesions are more likely to develop. Compared with *S. mutans*, *S. sobrinus* exhibits stronger acidogenicity and acid tolerance, and its ability to aggregate into dental-plaque biofilms via dextran-mediated interactions is also more pronounced. Rather than acting on a single pathway, SsNrtR appears to leverage NAD^+^ flux to simultaneously boost acidogenesis, strengthen biofilm cohesion, and tune viscoelastic behavior. Deleting nrtR cripples biofilm assembly and acid production, underscoring the central role of SsNrtR-controlled NAD^+^ metabolism in *S. sobrinus* virulence (Figure 9) These findings carry direct clinical weight: drugs aimed at dismantling biofilms could inadvertently perturb NAD^+^-dependent circuits, either delaying natural clearance or paradoxically reinforcing the community. Consequently, anti-biofilm therapies must be designed with a fine-grained understanding of such metabolic control hubs. Building on these observations, we plan to dissect the precise molecular links between *Ss*NrtR-regulated NAD^+^ metabolism and *S. sobrinus* pathogenicity in future work, and to explore whether the synergy between *S. sobrinus* and *S. mutans* is linked to NAD^+^ metabolism, thereby offering new strategies for dental caries prevention and control.

Despite the mechanistic insights provided, several limitations of this study should be acknowledged. The experimental findings were derived primarily from in vitro assays and a rodent caries model, which may not fully recapitulate the ecological complexity of the human oral environment; therefore, the external validity of these conclusions should be interpreted with caution. In addition, potential biological and environmental confounders—such as inter-individual differences in host immune responses, dietary carbohydrate exposure, and polymicrobial biofilm interactions—were not systematically controlled and may influence the functional contribution of SsNrtR in vivo. Future studies incorporating clinically relevant models and controlled microbial ecosystems will be essential to further substantiate the translational significance of this metabolic regulatory pathway.

## 5. Conclusions

This study establishes that SsNrtR-mediated regulation of NAD^+^ homeostasis is indispensable for the metabolic fitness and cariogenic phenotype of *Streptococcus sobrinus*. Genetic abrogation of *ssnrtR* resulted in impaired growth, diminished acidogenic capacity, and compromised biofilm architecture, leading to attenuated virulence in vivo. These findings identify SsNrtR as a mechanistically validated target with potential translational relevance for the development of anti-caries interventions.

## Figures and Tables

**Table 1:** Allocation of linear units to each molar.

Caries Site	Molars					
	Mandibular			Maxillary		
	1st	2nd	3rd *	1st	2nd	3rd *
Buccal	7	5	4	7	4	4
Lingual	6	5	4	6	3	4
Sulcal	8	6	3	6	3	3
Proximal	2 **	1	1	2 **	1	1

* 1st, 2nd and 3rd denote the first, second and third molars, respectively; ** The second molar contains a near-middle neighborhood and a far-middle neighborhood.

**Table 2:** Comparing caries severity (as measured by Keyes scores) among rat groups.

Group	Keyes Score (Caries Level)			
	E	Ds	Dm	Dx
WT	18.17 ± 0.89	8.32 ± 3.29	3.10 ± 1.51	2.11 ± 0.93
Δ*nrtR*	10.31 ± 2.01 *	4.35 ± 0.55 **	0.91 ± 0.62 **	0.23 ± 0.31 *
CΔ*nrtR*	15.96 ± 2.21	7.18 ± 2.08	2.63 ± 1.02	0.62 ± 0.21

Dm, moderate dentinal penetration; Ds, slight dentinal penetration; Dx, extensive dentinal penetration; E, enamel only * *p* < 0.05 compared with the control group; ** *p* < 0.01 compared with the control group.

## Data Availability

All data has been included in the manuscript.

## Supplementary Materials

The following supporting information can be downloaded at: https://www.mdpi.com/article/10.3390/pathogens14121213/s1, Supplementary Material S1: Table S1: Strains used in this study; Table S2: Plasmids used in this study; Table S3: Primers used in this study; Supplementary Material S2: Supplementary Methods: Rat Caries Induction, Keyes Scoring.
