# Supplementary material for: Regulation of NAD+ Homeostasis by SsNrtR in Streptococcus Sobrinus: A Critical Determinant of Its Cariogenic Potential
Source: Pathogens. 2025 Nov 28;14(12):1213. doi: 10.3390/pathogens14121213 (PMC12735969; doi:10.3390/pathogens14121213)
Supplement: Supplementary file 1 [file pathogens-14-01213-s001.zip › Supplementary Material S1.pdf]

Supplementary Materials

Supplementary tables

Table S1 Strains used in this study

**Strains**	Relevant characteristics	Origins
E. coli DH5α	A cloning host	Lab stock
E. coli BL21(DE3)	Protein expression host	Lab stock
BL21-pET-*nrtR*	BL21(DE3) with pET28a-*nrtR*	This work
WQ125	Δ*nrtR* mutant of *S. sobrinus*	This work
WQ129	The complemented strain of Δ*nrtR* (CΔ*nrtR*)	This work
WQ132	The wild-type strain of *S. sobrinus* carrying pVA838-*lacZ*	This work
WQ133	The Δ*nrtR* mutant of *S. sobrinus* carrying pVA838-*lacZ*	This work
WQ134	WQ129 (CΔ*nrtR*) carrying pVA838-*lacZ*	This work
WQ135	The *S. sobrinus* strain with pVA838-P*pnuC-lacZ*	This work
WQ139	W125 (Δ*nrtR*) carrying pVA838-P*pnuC-lacZ*	This work
WQ140	WQ134 (CΔ*nrtR*) carrying pVA838-P*pnuC-lacZ*	This work
WQ142	*S. sobrinus* strain with pVA838-P*pncB-lacZ*	This work
WQ143	W125 (Δ*nrtR*) carrying pVA838-P*pncB-lacZ*	This work
WQ145	WQ134 (CΔ*nrtR*) carrying pVA838-P*pncB-lacZ*	This work

Table S2 Plasmids used in this study

**Plasmids**	Relevant characteristics	Origins
pET28a (+)	T7-driven expression vector, Km^R	Novagen
pET28a-*nrtR*	*nrtR* in NdeI and SalI sites of pET28a	This work
pVA838-*nrtR*	pVA838 carrying the *S. sobrinus nrtR* gene	This work
pVA838-*lacZ*	*LacZ* in SphI and SalI sites of pVA838	This work
pVA838-P*nrtR*-*lacZ*	*LacZ* with the promoter of *nrtR in* BamHI and SphI sites of pVA838	This work

Table S3 Primers used in this study

Primers	Primer sequences (5'--3’)
SsnrtR-F	GGAATTCCATATGATGAAAGCAAAGGAACGTCGTC
SsnrtR-R	ACGCGTCGACTTAATTATTAAAGACGATATGCTCC
nrtR-U-F	ACATGCATGCAAAAGGCA CAAGTTCCAATTGTG
nrtR-U-R	ACGCGTCGACCATGATTGCT GCTCCTTTACAGCA
nrtR-D-F	CGCGGATCCTGTGCTAAAGGGGCCAGTCCGTAT
nrtR-D-R	CGGGGTACCCTCCTTTAACTAAGATTGCAAGATA
CniaX-F	GGTGGTCAGATCTTAGATGTGTGGTCGTAT
CniaX-R	AAGAAATCTCCTTGTTACCCAAAACTAATAA
pncB-F	CGCAACATCTAAGAGGAAATCTTAGAGTTCTTA
pncB-R	CAACGGTGGTCCTCGCTTCTAACGTACTTATCA
pnuC-F	GGAACCGTGGAAGGTTGGTCTTCTACTATCCG
pnuC-R	CAACATCTAAGATCCACCCACCGTTACTCCACC
