# Supplementary material for: Regulation of NAD+ Homeostasis by SsNrtR in Streptococcus Sobrinus: A Critical Determinant of Its Cariogenic Potential
Source: Pathogens. 2025 Nov 28;14(12):1213. doi: 10.3390/pathogens14121213 (PMC12735969; doi:10.3390/pathogens14121213)
Supplement: Supplementary file 1 [file pathogens-14-01213-s001.zip › Supplementary Material S2.pdf]

2.10.1 In vivo Caries Model in Rats: Induction by Cariogenic Challenge

Rats were assigned random numbers generated using Microsoft Excel and subsequently allocated to groups based on these numbers. Each group was housed in individually ventilated cages (IVCs) under specific pathogen-free conditions. The experimental rats were subjected to a diet known to induce caries, following the dietary regimen established by Keyes in 2000(Jiangsu Xietong Pharmaceutical Bio-engineering Co., Ltd, Nanjing, China), with the exception of the control group rats. Antibiotics (ampicillin, chloramphenicol, and carbenicillin) were administered continuously for five days at concentrations of 1.0 g/kg in the diet and 1.0 g/L in the drinking water to deplete the natural oral microbiota. Endogenous Streptococcus sobrinus was confirmed to be eradicated through oral sampling. Oral swab samples were cultured on Mitis salivarius agar plates (Difco) for 48 hours to confirm bacterial eradication. A sterile cotton swab was used to spread 1 mL of bacterial mixture on the surface of the upper and lower molars of the rats, which were then fasted within 1 hour after they spread. The rats were continuously infected with S. sobrinus for seven days. Over the next four days, rats were infected with S. sobrinus at a concentration of 1×10^8 CFU/ml to promote the colonization of cariogenic bacteria. Swab samples were then inoculated onto MSB plates to confirm S. sobrinus infection. Subsequently, rats were orally infected with fresh S. sobrinus cultures via daily oral swabs for four consecutive days. On day 5 post-infection, oral swabs were collected and inoculated onto Todd Hewitt agar plates, which were incubated at 37°C in a 5% CO_2 atmosphere to confirm colonization. On day 35 after bacterial inoculation, the rats were placed in a euthanasia chamber, the chamber was sealed, and CO_2 was administered through the gas inlet at a rate of 10–30% of the chamber volume per minute. Once the rats lost consciousness and ceased movement, the gas flow was increased to a maximum of 0.5 kPa. The CO_2 flow was stopped only after the rats became immobile, their respiration ceased, and their pupils dilated. The rats were then observed for an additional 2 minutes to confirm death.

According to the Keyes caries scoring system, caries are classified into four grades: enamel caries (E), mild dentinal caries (Ds), moderate dentinal caries (Dm), and extensive dentinal caries (Dx). Ds-grade caries indicates that the caries affect one-quarter of the dentin from the enamel to the pulp chamber floor; Dm-grade caries represents caries progression to one-quarter to three-quarters of the dentin between the enamel and the pulp chamber floor, while Dx-grade caries is marked when caries affect three-quarters of the dentin. The total E-grade caries score for the smooth surfaces of all molars is calculated by summing the individual scores for E-grade caries, and similar scoring methods are used for fissure E, Ds, Dm, and Dx-grade caries. The scoring process was single-blinded, and caries scores were determined by one calibrated examiner and two additional examiners.

2.10.2 The ethical animal maintenance and health surveillance

To safeguard animal welfare and ensure experimental rigor, all procedures were designed to minimize pain, distress, and suffering. Postsurgical analgesia was administered as required, and animals were consistently monitored for behavioral or clinical signs of discomfort. Any adverse events were documented and promptly managed with veterinary support. Predefined humane endpoints based on specific indicators of severe distress or morbidity were strictly

observed to avoid unnecessary suffering, with monitoring frequency tailored to individual animal health.

Rats were maintained under specific pathogen‑free (SPF) conditions in a controlled environment with a 12/12-hour light/dark cycle, ambient temperature of 20‑26 ° C, and relative humidity of 40% ‑ 70%. They received a specialized pellet diet and water ad libitum. Environmental enrichment was provided to support natural behaviors and well-being. All housing conditions were regularly audited and met the standards stipulated by the Animal Ethics Committee.These measures collectively upheld the highest standards of animal welfare and scientific integrity throughout the study.
